# Assessing the individual risk of stroke in caregivers of patients with stroke

**DOI:** 10.1055/s-0044-1779691

**Published:** 2024-03-11

**Authors:** Juan Manuel Marquez-Romero, Jessica Romo-Martínez, Bernardo Hernández-Curiel, Angélica Ruiz-Franco, Rita Krishnamurthi, Valery Feigin

**Affiliations:** 1 Instituto Mexicano del Seguro Social, Órgano de Operación Administrativa Desconcentrada, Hospital General de Zona #2, Departamento de Neurología, Aguascalientes AGS, Mexico.; 2 Instituto Mexicano del Seguro Social, Órgano de Operación Administrativa Desconcentrada, Centro Médico de Occidente, Departamento de Radiología, Guadalajara JAL, Mexico.; 3 Hospital Hispano Americano, Departamento de Neurología, Mexicali BC, Mexico.; 4 Hospital Juárez de México, Departamento de Neurología, Ciudad México CDMX, Mexico.; 5 National Institute for Stroke & Applied Neurosciences, School of Clinical Sciences, Auckland AUK, New Zealand.

**Keywords:** Stroke, Risk Factors, Caregivers, Accidente Cerebrovascular, Factores de Riesgo, Cuidadores

## Abstract

**Background** Genetic factors influence the risk of developing stroke. Still, it is unclear whether this risk is intrinsically high in certain people or if nongenetic factors explain it entirely.

**Objective** To compare the risk of stroke in kin and nonkin caregivers.

**Methods** In a cross-sectional study using the Stroke Riskometer app (AUT Ventures Limited, Auckland, AUK, New Zealand), we determined the 5- and 10-year stroke risk (SR) among caregivers of stroke inpatients. The degree of kinship was rated with a score ranging from 0 to 50 points.

**Results** We studied 278 caregivers (69.4% of them female) with a mean age of 47.5 ± 14.2 years. Kin caregivers represented 70.1% of the sample, and 49.6% of them were offspring. The median SR at 5 years was of 2.1 (range: 0.35–17.3) versus 1.73 (range: 0.04–29.9), and of 4.0 (range: 0.45–38.6) versus 2.94 (range: 0.05–59.35) at 10 years for the nonkin and kin caregivers respectively. In linear logistic regression controlled for the age of the caregivers, adding the kinship score did not increase the overall variability of the model for the risk at 5 years (R ^2^ = 0.271; *p* = 0.858) nor the risk at 10 years (R ^2^ = 0.376; *p* = 0.78).

**Conclusion** Caregivers of stroke patients carry a high SR regardless of their degree of kinship.

## Resumen

**Antecedentes** Los factores genéticos probablemente influyen en el riesgo de desarrollar enfermedad vascular cerebral (EVC), pero no está claro si el riesgo es intrínsecamente alto o si es totalmente explicado por factores modificables.

**Objetivo** Comparar el riesgo de EVC (REVC) en cuidadores pertenecientes y no pertenecientes a la misma familia de pacientes con EVC.

**Métodos** En un estudio transversal que utilizó la aplicación Stroke Riskometer (AUT Ventures Limited, Auckland, AUK, Nueva Zelanda), determinamos el REVC a 5 y 10 años en cuidadores de pacientes hospitalizados por EVC. El grado de parentesco se graduó con un puntaje de 0 a 50 dependiendo de su relación familiar con el paciente.

**Resultados** Estudiamos a 278 cuidadores (69.4% de ellos mujeres) con edad media de 47.5 ± 14.2 años. Los cuidadores familiares representaron el 70.1% de la muestra, siendo el 49.6% hijos. Las medianas de REVC a 5 años fueron de 2.1 (rango: 0.35–17.3) *versus* 1.73 (rango: 0.04–29.9), y de 4.0 (rango: 0.45–38.6) *versus* 2.94 (rango: 0.05–59.35) a 10 años para el grupo de cuidadores familiares y no familiares, respectivamente. En una regresión logística lineal contralando para la edad de los cuidadores, la adición del puntaje de parentesco no incrementó la variabilidad general del modelo para el riesgo a 5 años (R ^2^ = 0.271; *p* = 0.858) ni para el riesgo a 10 años (R ^2^ = 0.376; *p* = 0.78).

**Conclusión** Los cuidadores de pacientes con EVC tienen un REVC alto, independientemente de su grado de parentesco.

## INTRODUCTION

Both the incidence and mortality rates regarding stroke are consistently decreasing in high-income countries (HICs). Still, in low- and middle-income countries (LMICs), the number of people who die or remain disabled due to stroke has increased significantly over the last three decades. 1 Additionally, between 70% and 87% of all deaths and cases of disability derived from stroke occur in LMICs, where the burden of stroke is increasing particularly fast. 2

Although the prevalence of risk factors for the development of stroke is well described in HICs, the epidemiology for the same data in LMICs is scarce. 3 In response to these shortcomings, in recent years, regional initiatives have been put in place to tackle the increasing burden of stroke. 4 Despite these efforts, implementing effective, low-cost strategies for primary prevention is still a significant challenge. 5

Caregivers of patients with stroke and coronary heart disease are a subpopulation with a particularly elevated cardiovascular risk, mainly attributed to the emotional stress derived from the burden of care. Unlike HICs, where patients receive care in specialized facilities, the burden of care in Latin America resides in kin caregivers and occurs mainly in the family household. 6

Since both patient and caregiver have a shared environment, there may be differences in the stroke risk (SR) in caregivers with different degrees of kinship. To explore this hypothesis, the present project aimed to compare the SR of kin and nonkin caregivers of stroke patients determined using the Stroke Riskometer app (AUT Ventures Limited, Auckland, AUK, New Zealand).

## METHODS

### Participants

For the present cross-sectional study, we recruited participants from the inpatient clinics of three public hospitals in Mexico. During daily neurology rounds, we identified the caregivers of the stroke patients. Those who consented to participate were further interviewed.

To estimate the SR, we used the Stroke Riskometer app, a validated tool to prevent and predict the SR developed by the National Institute for Stroke & Applied Neurosciences of the Auckland University of Technology (AUT), in New Zealand. 7 It is based on the Framingham cohort, but has been improved by the inclusion of novel and recently-identified SR factors. 8 Using self-reported information, the app calculates the 5- and 10-year absolute risk of stroke for a given individual compared with the risk among the general population without the SR factors reported by the user. The information required includes age, sex, height, weight, ethnicity, arterial pressure, diabetes mellitus, hypertension, cardiovascular disease, cognitive deficit, traumatic brain injury, and family history of stroke. The app has been translated into 19 of the world's most spoken languages, and subsequent studies have demonstrated its transcultural validity in multiple countries from Africa to Latin America. 9 In the present study we used the Spanish version of the app.

After the participants filled out the app questionnaire, we recorded the values for the estimated risk along with questions regarding whether the participant shared the household, the diet, or both. Then, kinship was assigned a numeric score by each participant. Kinship scores were based on the average percentage of DNA shared between relatives: 50 for parents, offspring, and siblings; 25 for grandparents, uncles, aunts, nephews, and nieces; 12.5 for cousins; and 0 for spouses and other nonkin caregivers. 10

The caregiver status was established when the participants were interviewed. The interviews occurred during the first week of the in-hospital stay of the stroke patient.

### Statistical analysis

We collected, codified, and captured all the information in an electronic database. The categorical variables were expressed as frequencies and percentages. For comparisons among groups, we used the Chi-squared test. The Shapiro-Wilk test was used to test for the normality of the continuous variables. None of the collected variables showed a normal distribution. Therefore, all continuous data were presented as median (minimum–maximum) values. Differences among groups were determined through the Mann-Whitney U test and the independent samples Kruskal-Wallis test. The Spearman correlation coefficient was calculated to determine the correlations regarding the variables.

To examine the effect of the kinship score on the SR, we performed a two-step hierarchical linear regression, which included a first model with the age of the caregiver and a second model in which we added the kinship score. R values and β coefficients with 95% confidence intervals (95%CIs) were reported. For all analyses, we considered statistically significant values of *p* ≤ 0.05. All analyses were performed using the IBM SPSS Statistics for Windows, version 22.0 (IBM Corp., Armonk, NY, United States).

## RESULTS

We analyzed data from 278 caregivers, 193 of whom were women (69.4%). The mean age of the sample was of 47.5 ± 14.2 years. Most were kin caregivers (195; 70.1%). In order of frequency, the degrees of kinship were as follows: offspring (49.6%), spouses (23.4%), siblings (14.4%), other kin (6.2%), and parents (2.9%).On the other hand, nonkin caregivers were mainly spouses (23.4%), other nonkin (3.6%), and sons-in-law (2.9%).

The median body mass index (BMI) was of 27.2 (range: 14.8–44.1) Kg/m ^2^ , and the frequency of obesity (BMI ≥ 25 Kg/m ^2^ ) was of 69.8%. A shared household was reported by 44.6% of all the caregivers, and 61.2% reported a shared diet with the stroke patient. The prevalence of SR factors was of 33.5% for smoking, of 3.6% for alcohol intake, of 28.4% for hypertension, of 18.3% for diabetes mellitus, and of 6.5% for cardiovascular disease. During the interview, 37.1% of the caregivers reported adopting a diet that complied with international recommendations regarding the intake of fruits and vegetables, 40.3% reported exercising at least 2.5 hours a week, and 71.6% reported significant stress during the year before the interview. Table 1 shows the differences between the caregivers according to their kinship scores.

**Table 1:** Comparison of risk factors for stroke among groups of caregivers

Variable		Any kinship, *n* = 195	No kinship, *n* = 83	*p* value
Female sex: n (%)		137 (70.3)	56 (67.5)	0.642*
Age in years: median (minimum–maximum)		46 (20–85)	52 (20–80)	< 0.001**
Body mass index in Kg/m ^2^ : median (minimum–maximum)		27.3 (14.8–42.2)	26.9 (17.9–44.1)	0.328**
Obesity: n (%)		140 (71.8)	54 (65.1)	0.256*
Shared household: n (%)		57 (29.2)	67 (80.7)	< 0.001*
Shared diet: n (%)		99 (50.8)	71 (85.5)	< 0.001*
Stroke risk factors: n (%)	Smoking	65 (33.3)	28 (33.7)	0.936*
	Alcohol intake	8 (4.1)	2 (2.4)	0.491*
	Hypertension	54 (27.7)	25 (30.1)	0.683*
	Diabetes mellitus	33 (16.9)	18 (21.7)	0.366*
	Cardiovascular disease	8(4.1)	5 (6.0)	0.688*
	Healthy diet	69 (35.4)	34 (41.0)	0.381*
	Exercise	70 (40.0)	34 (41.0)	0.879*
	Emotional stress	138 (70.8)	61(73.5)	0.651*

Notes: *Chi-squared test. **Mann-Whitney U test.

The frequencies of the kinship scores were 83 caregivers (29.9%) with a score of 0, 186 (66.9%) with a score of 50, 6 (2.2%) with a score of 25, and 3 caregivers (1.1%) with a score of 12.5. The median (minimum-maximum) value for SR among all participants was of 1.75 (0.04–29.9) at 5 years, and of 3.2 (0.05–59.35) at 10 years. Table 2 shows the results of the SR estimation at five and ten years by the kinship score. The five-year score risk was similar among the groups, but at ten years, the risk was higher among non-kin caregivers; this difference was statistically significant. There were no differences in terms of risk in the subgroup analysis by kinship score. When compared by shared household, diet, or both, neither were there differences in terms of risk.

**Table 2:** Five- and ten-year estimated risk of stroke by kinship score

Variable	No kinship, *n* = 83	Any kinship, *n* = 195			*p* value
5-year risk: median (minimum-maximum)	2.1 (0.35–17.3)	1.73 (0.04–29.9)			0.055*
		12.5%; *n* = 3	25%; *n* = 6	50%; *n* = 186	
		2.8 (0.55–4.56)	2.2 (0.64–5.6)	1.7 (0.04–29.9)	0.300**
10-year risk: median (minimum-maximum)	4.0 (0.45–38.6)	2.94 (0.05–59.35)			0.047*
		12.5%; *n* = 3	25%; *n* = 6	50%; *n* = 186	
		4.3 (0.93–7.74)	3.2 (0.86–13.1)	2.9 (0.05–59.4)	0.252**

Notes: *Mann-Whitney U test. **Independent samples Kruskal-Wallis test.

In the correlation analysis, the 5-year values showed a non-significant negative correlation with kinship (Spearman Rho = - 0.115; *p* = 0.055). In contrast, the 10-year risk estimation showed a statistically significant negative correlation with the kinnship score (Spearman Rho = - 0.121, *p* = 0.044).

Lastly, as shown in Table 3 , the two-step hierarchical linear regression results demonstrated that the inclusion of the kinship score did not significantly contribute to increase the variability of the initial model controlled for the age of the participants; this was the case for both the five-and ten-year risk of stroke.

**Table 3:** Results of the two-step hierarchical linear regression

Variable		Adjusted R ^2^	∆R ^2^	β	95% confidence interval	*p* value
5-year risk	Model 1	0.273	0.276			
	Age			0.130	0.105–0.155	< 0.001
	Model 2	0.271	0.000			
	Kinship score			- 0.001	-0.017–0.014	0.856
10-year risk	Model 1	0.378	0.381			
	Age			0.343	0.291–0.395	< 0.001
	Model 2	0.376	0.000			
	Kinship score			0.005	-0.028–0.038	0.777

## DISCUSSION

To our knowledge, the present is the first study to explore differences in SR among caregivers with different degrees of kinship. Although our findings do not support a significant role of kinship in increasing the SR, this could be partially explained by the high prevalence of traditional SR factors among the studied population. The role of kinship might have been masked due to the magnitude of the strength of the association between traditional risk factors and stroke.

The degree of kinship directly correlates with genetic similarity of individuals as determined by the sharing of DNA inferred to be identical by descent, even a few hundred single-nucleotide polymorphisms can suffice to infer close familial relationships. 11 Therefore, the degree of kinship may play a role in the risk of stroke – a systematic review 12 with data up to 2018 identified at least 45 polymorphisms relevant to stroke pathophysiology. The identified polymorphisms have since been grouped in genetic risk scores and applied across different populations. In a study 13 involving 51,288 subjects with cardiometabolic disease, a genetic risk score was an independent predictor for ischemic stroke after adjusting for traditional risk factors (hazard ratio [HR]: 1.27; 95%CI: 1.04–1.53), especially among those with a low prevalence of traditional SR factors. In another study 14 involving 13,214 individuals, researchers described a polygenic risk score that was significatively associated with ischemic stroke (odds ratio [OR]: 1.75; 95%CI: 1.33–2.31); nevertheless, they clarified that their score should not be used independently but combined with the traditional risk factors for stroke.

Moreover, the shared environment between the caregivers and the patients with stroke further contributed to dissimulating the potential effect of kinship on the SR. Since 78.3% of the nonkin caregivers were the spouses of the patients, it would be more challenging to discern the contribution of critical environmental factors such as shared household and diet to the risk of stroke. Additionally, age, a nonmodifiable risk factor for stroke, was higher among nonkin caregivers.

Our study also shows a high frequency of traditional SR factors, particularly smoking (33.5%), hypertension (28.4%), and diabetes mellitus (18.3%). As in other societies, such as the Chinese, 15 the participants in the present study were mainly kin caregivers, especially their daughters. After removing the spouses, only 3.6% of the participants were nonkin caregivers. The high prevalence of SR factors is significant because of the known adverse effect on health-seeking behaviors (such as healthy diet, exercising, and stress management) observed among caregivers of patients with chronic disorders. It is also worth noting that, at the same time, unhealthy behaviors (such as smoking, alcohol consumption) increase among caregivers. 15 Although nearly 40% of the participants reported healthy habits (regular exercise and intake of fruit and vegetables within the international recommendations), 3 out of 4 participants reported being exposed to significant emotional stress the year before their interview. Adding the potential increase in perceived stress due to their new duties as caregivers of a patient with a stroke-related disability, a further rise in unhealthy and a decrease in healthy behaviors on the part of the caregivers is foreseeable, which will translate into a higher risk of stroke. Hence, the importance of making the caregivers aware of their increased risk by employing tools such as the Stroke Riskometer app.

We acknowledge the implicit limitation of the transversal design of the present study, because to evaluate the risk of stroke in family members of stroke survivors to determine the relative contribution of environmental and genetic risk factors would require a cohort design. Then, within the cohort, we would need to assess the risk of stroke in kin caregivers from all the levels of kinship (scores of 50, 25, 12.5, and 0) living with the stroke survivor for various periods of exposure time. Nevertheless, even after acquiring such a cohort, it would be very hard (virtually impossible) to measure the exposure time reliably. Regrettably, we need to adequately measure the exposure time to the same environmental factors shared by stroke survivors and their caregivers to address the contribution of genetic factors. As aforementioned, the contribution of environmental factors to the risk of stroke is much more significant. Still, the present study provides valuable insights regarding the profile of cerebrovascular risk factors and the degree of kinship in a sample of caregivers of stroke patients. These data can contribute to the tailored design of primary prevention strategies aimed at this particular population at risk of stroke.

In conclusion, the results of the present study show an elevated prevalence of traditional risk factors for stroke in a sample of caregivers of patients with stroke. The increased prevalence, in turn, translates into a higher risk of stroke at five and ten years. The heightened risk in caregivers appears independent of the degree of kinship to the patient under their care.
